# Diversity of *Drosophila* egg patterning: The missing tools to explore embryonic axis formation

**DOI:** 10.3389/fcell.2025.1569318

**Published:** 2025-03-21

**Authors:** Helen L. Stott, Nir Yakoby

**Affiliations:** ^1^ Center for Computational and Integrative Biology, Rutgers, The State University of NJ, Camden, NJ, United States; ^2^ Department of Biology, Rutgers, The State University of NJ, Camden, NJ, United States

**Keywords:** CRISPR/cas9, *Drosophila*, model clade, EGFR signaling, axis formation

## Abstract

Focusing on selected model organisms to establish scientific communities and resources has greatly advanced our understanding of biological processes, including embryogenesis, and facilitated the translation of these data into developing human remedies. However, by restricting our research to a small number of model organisms, we risk overlooking the underlying mechanisms controlling animal diversity and speciation. Changes in cell signaling, protein compatibility, and genetic tinkering are often neglected due to the lack of molecular tools in non-traditional model organisms. The era of high-throughput genome sequencing, computational gene prediction, and emerging genome editing and imaging tools, offers an opportunity to explore novel mechanisms of organismal development and homeostasis. As we develop new model platforms, it is imperative to prioritize resources effectively. What criteria make an organism a “good” candidate for becoming a new model organism for exploring embryogenesis? The axis of the *Drosophila* embryo is set during eggshell patterning. Although species with a dorsal ridge exhibit dramatically different patterns of the dorsalization signal, epidermal growth factor receptor activation, compared to *Drosophila melanogaster*, the embryonic dorsal-ventral axis remains consistent. Despite the increasing number of sequenced fly species’ genomes, the experimental tools necessary to study these species are still lagging. Here, we emphasize the need to further develop genetic and molecular tools for studying nontraditional model organisms to understand complex processes like evolution of maternal contribution and correct embryonic body axis. We address current challenges in achieving these goals, such as genetic markers, selectable markers, and the efficiency of CRISPR/Cas9 mediated genomic editing.

## 1 Introduction

The use of model organisms has been essential in unraveling complex mechanisms of biological functions. The similarity of biological functions across living organisms allows the scientific community to leverage the experimental advantages of model organisms for studying questions in many fields: cell division, transcriptional regulation and profiling, cell signaling, signaling integration, protein translation, tissue morphogenesis, organogenesis, and models for human diseases (Wangler et al., 2017; Bertile et al., 2023). The importance of these organisms was recognized by the Human Genome Project, the seminal scientific achievement of the 20th century. In addition to sequencing the entire haploid genome of humans, model organisms, such as bacteria, yeast, worms, flies, and mice were also sequenced (Lander et al., 2001; Hood and Rowen, 2013). This project opened the genomics era, prompting a shift from the traditional practice of testing one gene at a time to a high throughput quantitative study of all genes at organismal and single-cell levels. This transition was aided by using simple organisms such as *C. elegans* and *Drosophila melanogaster*, in addition to mice, as effective models for human diseases. Most research resources are still invested in a few model organisms that allow us to deeply investigate cellular components in the large puzzle of embryogenesis. However, some changes do not depend on a single modification, which requires the exploration of evolutionary changes in the native species. However, we still lack efficient molecular and genetic tools to study them.

## 2 Model organisms are useful and important tools for scientific research

Model organisms such as *Neurospora*, *C. elegans*, *D. melanogaster*, *Arabidopsis*, and *Mus musculus*, successfully established large communities to study metabolism, cell lineage and differentiation, signaling pathways and patterning, plant biology, and disease (Davis, 2004; Irion and Nusslein-Volhard, 2022; Jacob and Monod, 1961; Sinclair et al., 1998; Phifer-Rixey and Nachman, 2015; Victor Atoki et al., 2025). The fundamental similarities of basic processes across organisms permit the lateral transfer of information across distant organisms and consequently facilitates the translation of this knowledge to fight human pathologies. Traditional mutation screens generated thousands of mutant lines associated with various phenotypic groups (Nusslein-Volhard and Wieschaus, 1980; Schupbach and Wieschaus, 1986; Jorgensen and Mango, 2002; St Johnston, 2002; Mullins et al., 1994). Some of these mutations were in the same genes and others in genes that participate in the same process (i.e., axis formation, organogenesis, segmentation, etc.). These large-scale screens were bed-rock initiatives which created model-organism centered communities of scientists producing large bodies of work mechanistically connecting these phenotypes to genes.

## 3 Limitations of current model organisms

Model organisms have been essential to discovering a multitude of fundamental processes common in animals. For example, morphological and patterning changes can be simply explained by alteration in *cis*-regulatory modules. Examples include the fish egg-spot, digit number, wing spots, and limbs, to mention a few (Carroll, 2008; Carroll et al., 2008; Beldade and Brakefield, 2002; Santos et al., 2014; Lettice et al., 2008; Xu et al., 2020; Sagai et al., 2005). At the same time, evolution has tinkered with these mechanisms, adjusting species to exhibit unique functions. For example, the expression pattern of neuronal ectoderm (NEE) genes in flies’ embryos occupy the same ventral-lateral position across species. Reporter genes driven by the NEE enhancers successfully recapitulated the endogenous pattern in the corresponding native species. However, when the same reporters were expressed in *D. melanogaster*, they showed a large deviation from the ectoderm domain (Crocker et al., 2008). These findings display how co-evolving cellular environments maintain correct axis patterning, however, a single component, such as an enhancer, cannot fully account for the conserved domain across species.

The emergence of genome engineering tools, like CRISPR/Cas9, have reduced the need for substantial mutation screens in traditional model organisms (Bier et al., 2018; Biering et al., 2022; Kramer et al., 2018; Xu et al., 2017) and opened up a new avenue for more faithfully representing the cellular environments of species of interest. This allows the traditional approach of exploring one component at a time in a heterologous organism to be replaced by a new approach that allows us to explore the co-evolution of multiple components, some of them as yet unidentified (Cooper, 2024). Despite readily available genomes for many related species, both the computational methods and the genetic tools for conducting mechanistic studies outside of model organisms are limited (Tomoyasu and Halfon, 2020).

Notably, the common practice of using traditional model organisms to test functions of regulatory DNA or proteins from closely related species may conceal the actual interacting partners in the native species. Furthermore, testing functions in model organisms that are too evolutionary distant may lead to conclusions that do not automatically reflect their native function, but rather their function in the model organism. Phenotypes obtained through perturbation reflect the impact (sufficiency/necessity) of this mutation in the model organisms (Cooper, 2024).

As we work to explore developmental complexity and identify new mechanisms underlying species-specific traits, molecular tools should be expanded and adjusted for use in additional organisms. This broader approach will help to capture the scope of co-evolutionary processes across multiple levels (molecular, cellular, tissue, organismal, community). Additionally, deviation from focusing on a handful of traditional model organisms would facilitate the exploration of novel mechanisms in cell signaling, development, and homeostasis, helping us to better understand pathologies and find new strategies for curing diseases.

## 4 What makes an organism a good model?

Mendel’s research pioneered the field of genetics. His selection of peas as a model system was viewed unfavorably at the time and perceived as an isolated example. Yet, selecting a system that is easy to grow, has a short life cycle, produces many offspring by controlled crossing, and has discrete and easily identifiable true bred phenotypes made peas an excellent model organism for exploring the laws of inheritance (Stenseth et al., 2022). Similar rules apply for the discovery of the Lac-operon. A short generation time, a large number of progeny for finding rare mutations, no dependance on lactose for growth, and inducible genes, all made bacteria an excellent system to study the coordinated regulation of genes that participate in the same metabolic process (Jacob and Monod, 1961). Hence, the selection of a model system is essential once the research question is defined.

The successful path from model system to “true” model organism rests on building large and collaborative communities to develop robust and sophisticated resources, including molecular, genetic, and genomic tools, strategies for maintaining and sharing stocks, the creation and maintenance of databases, and the advancement of computational and bioinformatics tools (Irion and Nusslein-Volhard, 2022). A crucial first step in selecting new model organisms is leveraging the information and resources of an already established community. This can save time and effort, as genome engineering tools can be adapted to operate in related species with relatively modest adjustments. This approach opens new opportunities for exploring evolutionary tinkering in embryogenesis.

## 5 Focusing on *Drosophila*

Several productive communities have been working with a few model organisms. In this perspective, we focus on *Drosophila*, which has been central to breakthrough discoveries in developmental biology, cell signaling, neurobiology, computational modeling and bioinformatics, evolution, and genetics, to name a few (Victor Atoki et al., 2025). Research utilizing *D. melanogaster* embryos has cemented the connections between genomic and phenotypic change and provided countless translational insights into human diseases (Verheyen, 2022; Perrimon et al., 2016). From Morgan’s white-eyed phenotype (Morgan, 1910), through Muller’s X-ray mutation screens on the X chromosome (Muller, 1928), the large screens in Heidelberg (Nusslein-Volhard and Wieschaus, 1980; Schupbach and Wieschaus, 1986; Wieschaus and Nusslein-Volhard, 2016), the discovery of Hox genes (Lewis, 1978; McGinnis et al., 1984), the investigation of immune response (Hoffmann, 2007), and the elucidation of and circadian clock (Allada et al., 1998), *Drosophila* has been at the forefront of embryogenesis studies. These seminal discoveries have been recognized by the international community with numerous awards including six Nobel prizes (Mohr, 2018).

Most resources have been directed at *D. melanogaster* with some investment in other *Drosophila* species, including comparative genomics, evolution of cell signaling, patterning, morphologies, vision, behavior, and communication. However, the tremendous diversity among *Drosophila* species provides an unparalleled opportunity to study the nature of speciation *in vivo*, accounting for the complex co-evolving systems unique to each species. We argue that the lack of effective genome engineering tools prevents researchers in the *Drosophila* research community from fully leveraging this co-evolutionary wealth (Tomoyasu and Halfon, 2020). The combination of short life cycles, complex tissue organization with low genetic redundancy, sequenced genomes across multiple species, availability of species with novel patterning and morphologies, ease of quantitative imaging in both live and fixed samples, as well as advances in molecular and genome engineering tools, all make the *Drosophila* group an exciting “model clade” for exploring mechanisms underlying evolutionary changes (Kim et al., 2021; Brand and Perrimon, 1993; Niepielko et al., 2011; Niepielko et al., 2014; Kanca et al., 2022; Garcia et al., 2013). Hence, the conditions have ripened to systematically explore co-evolving circuits at both molecular and organismal levels.

## 6 A comprehensive example: EGFR signaling and embryo axis formation

The dorsal-ventral (DV) axis in *Drosophila* is established during oogenesis by the epidermal growth factor receptor (EGFR) (Roth, 1993). Specifically, in egg chambers, the precursor of the mature egg, the TGF-alpha like ligand Gurken (GRK), localized around the oocyte nucleus, activates EGFR in the overlaying follicle cells after being secreted into the perivitelline space (Nilson and Schupbach, 1999). During DV axis formation, EGFR signaling represses *pipe* (*pip*), limiting it to the ventral domain (Sen et al., 1998; Cho et al., 2010; Moussian and Roth, 2005). The ventral restriction of Pip confines the cleavage of the Toll pathway ligand, Spätzle, to the ventral side of the *Drosophila* embryo, thereby setting domains along the DV axis. Through *pip* regulation, the DV axis is highly sensitive to changes in EGFR activation levels; reduced levels ventralize the embryo by increasing the *pip* domain dorsally, and elevated levels dorsalize the embryos by reducing *pip* expression (Roth, 1993; Schupbach and Roth, 1994).

**FIGURE 1:**
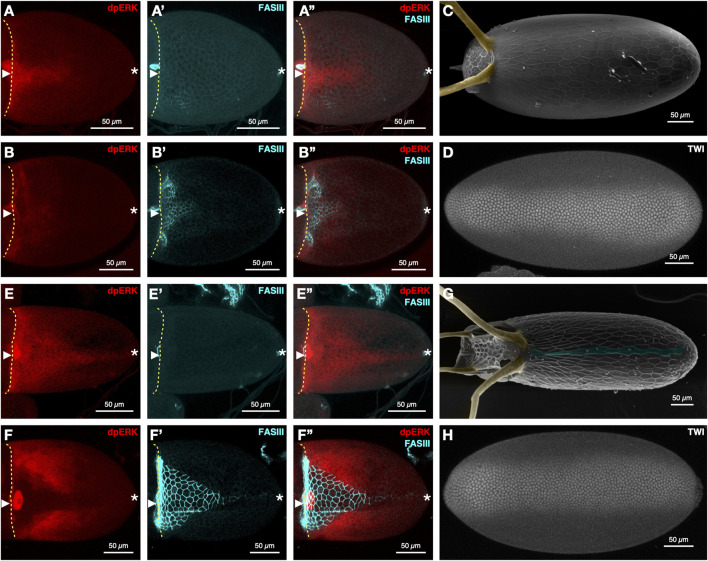
EGFR signaling and dorsal-ventral axis formation in *D. melanogaster* **(A–D)** and *D. nasuta* **(E–H)**. Stage 10A **(A-A”)** and 10B **(B-B″)** egg chambers of *D. melanogaster* stained for EGFR signaling (dpERK), FasIII, and merged (n = 7 and n = 28, respectively). *D. melanogaster* **(C)** eggshell (n = 12), dorsal appendages (yellow), and **(D)** embryo stained for Twist (n = 5). Stage 10A **(E-E″)** and 10B **(F-F″)** egg chambers stained for dpERK, FASIII, and merged (n = 12 and n = 9, respectively). *D. nasuta* **(G)** Eggshells (n = 27). Dorsal appendages (yellow) and dorsal ridge (blue), and **(H)** embryo stained for Twist (n = 5). Immunohistochemistry, confocal imaging, and SEM imaging were performed as described before (Niepielko and Yakoby, 2014; Stevens et al., 2022) (anti-dpERK antibody, 1:100, Cell Signaling; anti-FASIII antibody, 1:100, DSHB 1D4). Rat anti-TWIST (1:1,000; gift from the Wieschaus lab) was used as in (He et al., 2014). Anterior to the left for all images, dorsal views for egg chambers and eggshells, ventral views for embryos. The yellow broken line denotes the anterior of the oocyte. Arrowhead denotes the dorsal midline. Asterisk denotes the posterior end.

The level and duration of EGFR signaling on the dorsal side of the egg chamber, is dramatically different among *Drosophila* species during DV axis determination (Niepielko et al., 2014; Niepielko and Yakoby, 2014; Kagesawa et al., 2008). In *D. melanogaster*, EGFR activation is initially restricted to the dorsal midline of egg chamber (Figure 1A). This signal expands laterally a few hours later by activation of EGFR through Spitz (Figure 1B). EGFR activation leads to the formation of two dorsal appendage primordia on either side of the dorsal midline, marked by fasciclin III (FASIII) (Figures 1A’, B’), which give rise to the two embryonic respiratory structures, the dorsal appendages (Figure 1C). The mesoderm, marked by Twist (Twi), is restricted to ∼30% of the cells in the ventral domain of the embryo (Figure 1D).

In *Drosophila* species with a dorsal ridge - a lumen-like structure on the embryo casing (Niepielko and Yakoby, 2014) - the level, duration, and domains of EGFR activation differ considerably from the pattern observed in *D. melanogaster*. In *D. nasuta*, EGFR signaling levels are substantially elevated along the dorsal side of the egg chamber during early stage 10A (Figure 1E). The memory of this activation pattern is reflected in the extended expression of FASIII at stage 10B (compare *D. melanogaster* Figure 1B’ to *D. nasuta* Figure 1F’). Additionally, the lateral expansion of EGFR signaling at stage 10B is more extensive in *D. nasuta* than *D. melanogaster* (compare *D. melanogaster* Figure 1B to D*. nasuta* Figure 1F) and contributes to the formation of four dorsal appendages in the final eggshell (Figure 1G).

**FIGURE 2:**
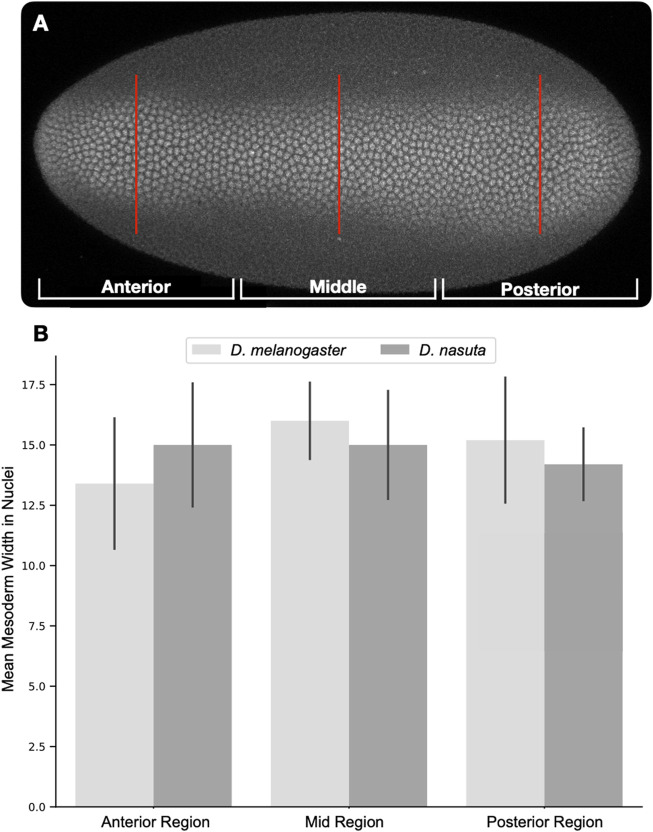
Quantification of mesoderm width using Twist positive nuclei. Dorsally oriented embryos were divided into thirds (anterior, middle, posterior). Twist positive nuclei were counted along a line in the center of each third [**(A)**, red lines]. Nuclei counts were averaged for each region and compared across both species [**(B)**, *D. melanogaster* n = 5, *D. nasuta* n = 5]. Comparison of nuclear counts at each region using the Student’s t-test revealed no significant differences.

Interestingly, despite the expansion of dorsal EGFR signaling in *D. nasuta* and the expected change in DV axis, the mesoderm of the *D. nasuta* embryo still spans the same number of cells as in *D. melanogaster* (Figures 1F, 2A,B). Furthermore, in *D. nasuta*, the dorsal anterior activation of EGFR expands laterally, yet the anterior mesoderm domain unexpectedly trends to span more nuclei (Figures 1D,H; Figure 2). Given the sensitivity of the embryos’ DV axis to changes in EGFR signaling (Queenan et al., 1997), it is intriguing to explore how the mesoderm maintains a consistent ventral domain despite the differences in EGFR signaling.

## 7 What’s next? How do we explore diverse EGFR activation levels with constant DV axis?

To account for the spatial changes in EGFR signaling in these species, molecular and computational tools must be employed to explore directly co-evolving components of EGFR signaling and downstream targets.

### 7.1 Experimental tools to explore casual effects

The long history of *D. melanogaster* as a prominent model organism for genetic research has led to the development of an extraordinary array of molecular and genetic tools to unravel complex processes in biology. Some of the key innovations include the use of dominant, homozygous lethal mutations and balancer chromosomes to track genetic manipulations and prevent recombination, the implementation of targeted expression systems like UAS/Gal4 for functional perturbations, the cultivation white-eyed flies allowing for easy screening of transgenics and the optimization of gene insertion techniques like the phiC31 integrase vector system and genome editing techniques like CRISPR/Cas9 (Brand and Perrimon, 1993; Kanca et al., 2022; Stern et al., 2023; Gratz et al., 2014). For example, we can insert coding and non-coding sequences from other *Drosophila* species into the *D. melanogaster* genome to test their effects on signaling intensity or gene expression. Inserting *grk* from *D. willistoni,* a dorsal ridge species, into *D. melanogaster* not only rescued a *grk* null fly, but also generated a short dorsal ridge in a low percentage of the eggshells (Niepielko and Yakoby, 2014).

Importantly, the same *grk* in *D. willistoni* is necessary for the robust formation of dorsal ridge, however, the penetrance in *D. melanogaster* is very low, likely due to other components unique to *D. willistoni* (Niepielko and Yakoby, 2014). Similar experiments can be done with *grk* from *D. nasuta* and *D. nebulosa*, as well as other components related to EGFR signaling pathway, including downstream targets like *rhomboid*, *spitz*, *sprouty*, and *kekkon* controlling both positive and negative feedback loops of signaling (Zartman et al., 2009; Shilo, 2005). However, testing one component at a time in *D. melanogaster* is time consuming, costly, and does not always reflect the full function of the gene in the native species. August Krogh noted in his 1929 essay that for a given question, there is usually a particular animal model best suited to address that question (Garcia et al., 2013). The most suitable system for answering coevolutionary questions is not a single model organism. Answering these questions requires the development of a “model clade,” a group of closely related species accompanied by the genetic and molecular tools necessary for reciprocal editing to more accurately examine predicted functions in the relevant context.

### 7.2 Challenges in manipulating other *Drosophila* species

CRISPR/Cas9 was used successfully to disrupt genes in other *Drosophila* species (Baker et al., 2024; Lamb et al., 2020; Sottolano et al., 2022), however, unlike its use in *D. melanogaster*, achieving homology directed repair (HDR) in non-model Drosophilids has been mostly unsuccessful with one exception (Stern et al., 2023). Additionally, the lack of balancer chromosomes and dominant genetic markers makes it difficult to follow and maintain edits to the genome. Most *D. melanogaster* transgenic flies rely on complementation of the *white* gene mutation, or the use of fluorescent eye markers to track successful integration of transgenes in a white-eyed fly (Bellen and Yamamoto, 2015). Despite this common practice, the disruption of the *white* gene affects courtship behavior and mating success in *D. melanogaster* (Xiao et al., 2017). Although white-eyed stocks have been established in other *Drosophila* species (Holtzman et al., 2010), in *D. nebulosa*, white-eyed males do not attempt to mate at all, preventing the effective use of white-eyed flies and fluorescent eye markers in this species (Sottolano et al., 2022). Likewise, mutations in the *Of-white* gene of the Milkweed bug *Oncopeltus fasciatus* were lethal (Reding and Pick, 2020).

Genome manipulations in *D. melanogaster* were not always simple and have undergone a series of optimizations. Incorporating Cas9 and phiC31 integrase under the control of germ line promoters greatly improved transgenesis efficiency in this species (Bischof et al., 2007; Kondo and Ueda, 2013). This same strategy has been used in a variety of non-model insects to improve gene editing outcomes (Li et al., 2017; Sun et al., 2017). Several labs have recently published methods for increasing HDR efficiency in *D. melanogaster*, including the use of short homology arms and the pJAT plasmid platform (Kanca et al., 2022; Stern et al., 2023). Incorporating a species-specific native *nos* promoter to drive *cas9* in the germline, together with the more efficient CRISPR strategies may expedite successful HDR in a variety of non-model *Drosophila* species. Options for maintaining the inheritance of edited loci are also emerging. A suite of transgenic tools developed by the Stern lab was used to generate a balancer chromosome for *D. simulans* (Stern, 2022). It may be possible to replicate this success in other non-model Drosophilids to stabilize transgenic constructs by preventing recombination.

Another recent innovation is the use of antibiotic resistance genes as selectable markers in flies (Matinyan et al., 2021), which eliminates the need to screen for visible selection markers and reliance on white-eyed fly strains. Growing transgenic flies on antibiotic treated food may permit the maintenance of deleterious edits across multiple generations without the use of balancer chromosomes. Additionally, titration of antibiotic concentrations could facilitate the selection of offspring homozygous for edits without the use of additional markers to follow the edited and unedited chromosomes through sequences of crosses. Another more traditional option is to identify non-vision related mutations on the X chromosome (like the *white* gene). The *Drosophila* wing has two cross-veins regulated by *crossveinless-2* (*cv-2*). Mutations in *cv-2* lead to the loss of the two cross veins on the wing, a simple visual phenotype (Conley et al., 2000). The *cv-2* cDNA (4329bp) is of similar size to the commonly used *mini-white* cassette (4136bp) in transformation vectors.

### 7.3 Computational tools to predict co-evolution

Beyond the ability to perturb known genes in a signaling pathway and their known targets in new model systems, we can also leverage computational predictions of co-evolving domains in interacting proteins to further refine our exploration of co-evolving systems. The sequenced genomes of flies like *D. nasuta and D. nebulosa* provide a good starting point for exploring how evolution along the EGFR signaling pathway can both maintain stasis for critical functions like mesoderm specification while also permitting the emergence of new forms like the dorsal ridge, a downstream target of elevated EGFR signaling (Niepielko et al., 2014; Niepielko and Yakoby, 2014). Phylogenetic comparative methods like iBIS2 (protein-protein co-evolution, (Oteri et al., 2022)) and PhyloACC (convergent evolution in regulatory elements, (Hu et al., 2019)) can highlight significant areas of convergence and divergence linked to selected phenotypic outcomes like the dorsal ridge.

One hurdle in implementing this approach is the lack of comprehensive collections of phenotype data, particularly data that identifies instances of phenotypic convergence, to match our growing library of high-quality genomes. Once implemented, labeling strategies like immunohistochemistry and Single Molecule Fluorescent *in situ* Hybridization can provide complimentary insights by revealing cross-species changes in gene expression patterns, protein localization, and signaling intensity. These methods can provide valuable spatiotemporal markers to further support the insights from phylogenies. Although these methods are still correlative, the generated predictions can be prioritized and validated in the new gene editing platforms, thus accounting for the overall evolutionary changes that occurred during speciation.

## 8 Concluding remarks

The triangle of a biological question, experimental tools to address the question, and computational tools to analyze the data have come to a full circle in the post-genomics era. High-quality genome sequences of many species, beyond the traditional model organisms, are available (Kim et al., 2021). Additionally, predictive computational tools to identify co-evolving domain are also accessible (Oteri et al., 2022; Hu et al., 2019). Therefore, the groundwork is in place to tackle the next challenge: understanding speciation in native species. To accomplish that, computational tools will need to be supplemented with species’ traits (e.g., morphology, pattern, function, etc.) to narrow down the list of predicted co-evolving domains. Moreover, adjusting genome engineering tools to enable HDR, together with robust and inexpensive selection of transgenic organisms will allow the scientific community to explore complex, heterogenous systems within their native context. We argue that branching out from established communities, like that of *Drosophila*, is the most efficient path forward.

## Data Availability

The raw data supporting the conclusions of this article will be made available by the authors, without undue reservation.
